# Supplementary material for: PP2A‐B56 binds to Apc1 and promotes Cdc20 association with the APC/C ubiquitin ligase in mitosis
Source: EMBO Rep. 2019 Dec 11;21(1):e48503. doi: 10.15252/embr.201948503 (PMC6945068; doi:10.15252/embr.201948503)
Supplement: Supplementary file 1 — Appendix [file EMBR-21-e48503-s001.pdf]

Appendix for

PP2A-B56 binds to Apc1 and promotes Cdc20 association with the APC/C ubiquitin ligase in mitosis

Kazuyuki Fujimitsu[1] and Hiroyuki Yamano[1*]

[1]*Cell Cycle Control Group, UCL Cancer Institute, University College London, London, WC1E 6DD, UK*

[*]Corresponding author. Email: h.yamano@ucl.ac.uk

Table of contents

Appendix Figure S1: Sequence alignment and location of Apc1-loop[500] in the N-terminal WD 40 domain of Apc1 page 2

A

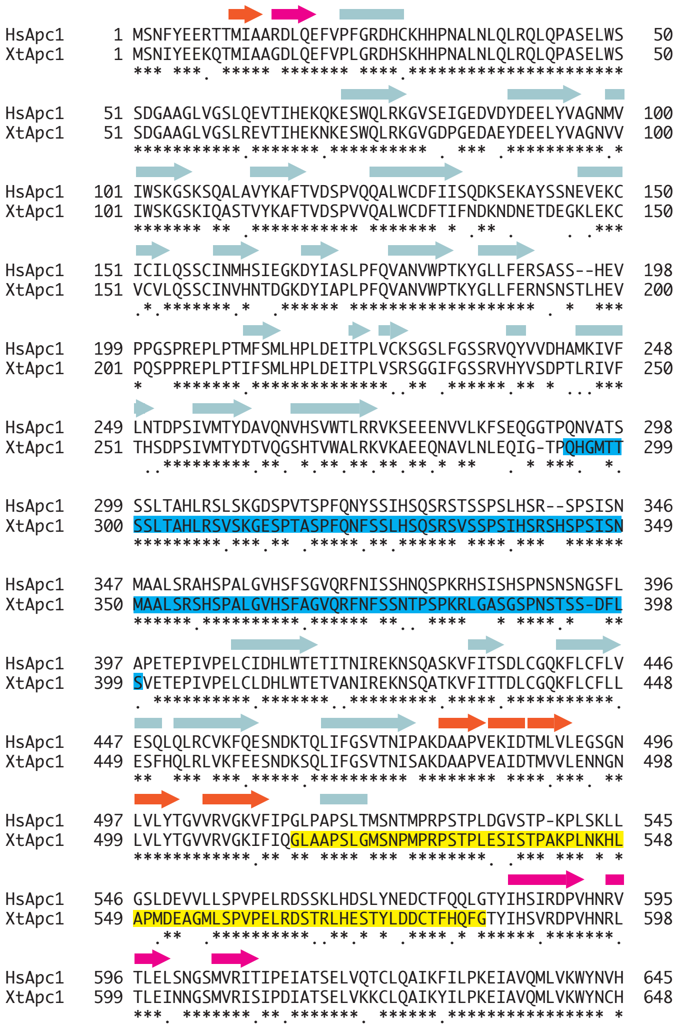

B

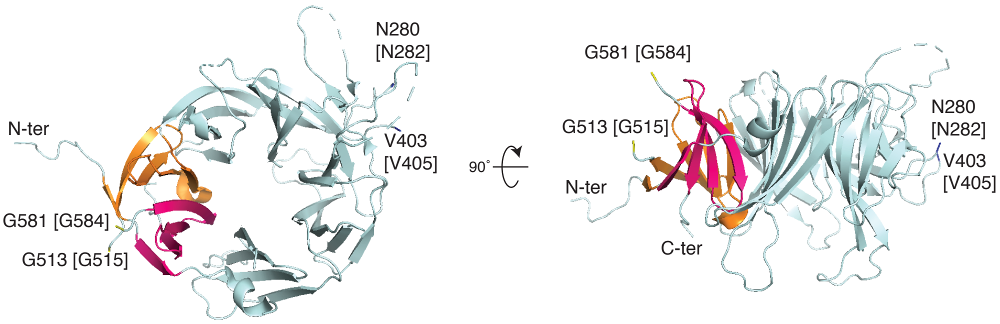

Figure S1. Sequence alignment and location of Apc1-loop500 in the N-terminal WD 40 domain of Apc1.

A. Sequence alignment of the N-terminal WD 40 domain of Homo Sapiens (Hs) Apc1 and Xenopus tropicalis (Xt) Apc1 is shown. According to a report from Barford lab [39], α-helix and β-sheets are shown above the sequences as block and block arrow, respectively. The sequences of Apc1-loop300 (Blue) and Apc1-loop500 (Yellow) of Xt Apc1 are highlighted. Apc1-loop500 is located between blade 6 (orange) and blade 7 (magenta).

B. The structural image of the N-terminal WD 40 domain is generated by Protein Data Bank file (5LGG: Human N-terminal WD40 domain of Apc1) and PyMOL, highlighting blade 6 (orange) and blade 7 (magenta). The number denotes the position of the amino acid residue of Apc1, first in human and then in Xenopus in square bracket.
