# Supplementary material for: PP2A‐B56 binds to Apc1 and promotes Cdc20 association with the APC/C ubiquitin ligase in mitosis
Source: EMBO Rep. 2019 Dec 11;21(1):e48503. doi: 10.15252/embr.201948503 (PMC6945068; doi:10.15252/embr.201948503)
Supplement: Supplementary file 2 — Expanded View Figures PDF [file EMBR-21-e48503-s002.pdf]

Expanded View Figures

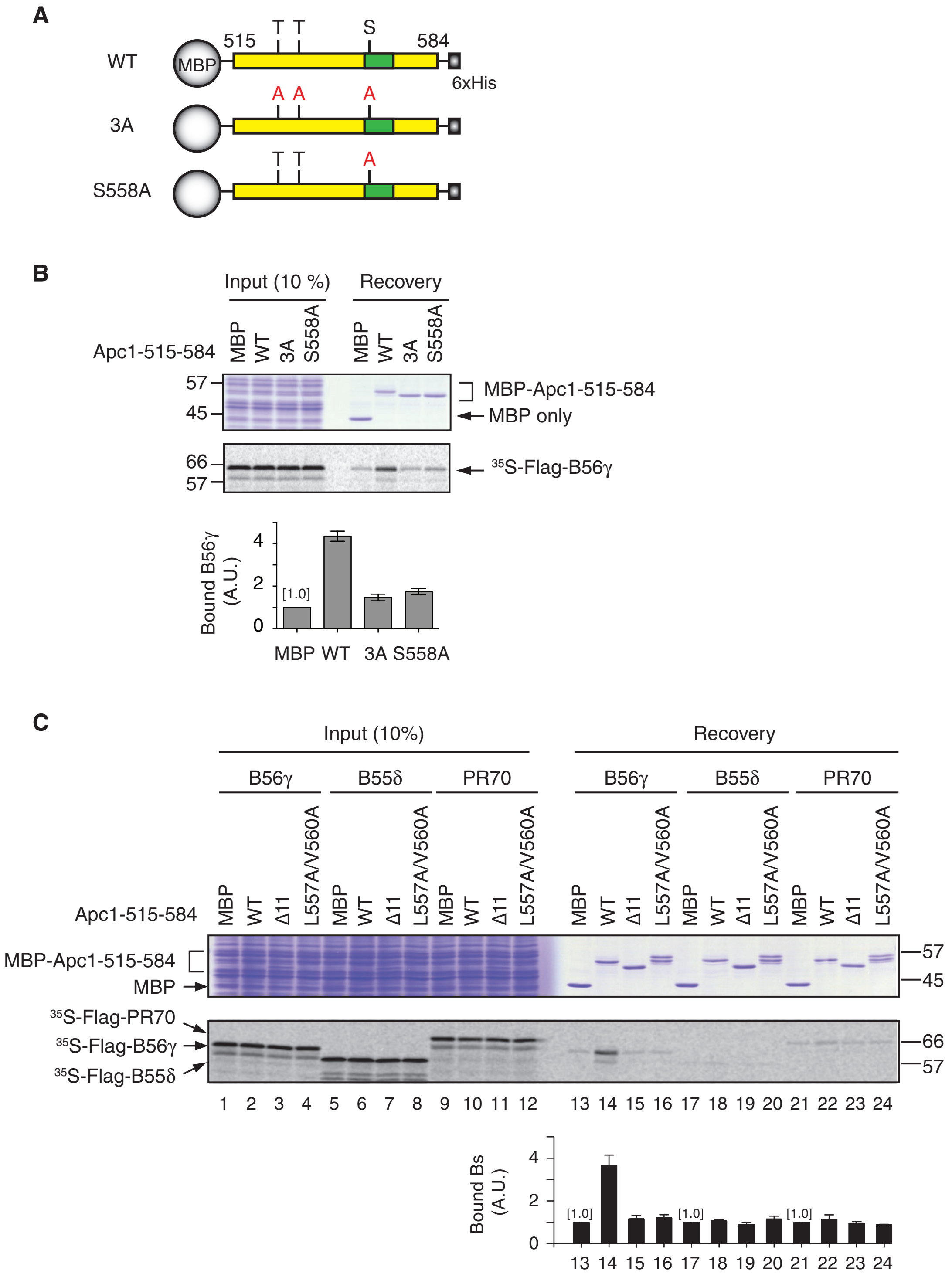

Figure EV1.

Figure EV1. Effects of mutations at Cdk sites of Apc1-loop^{500} towards B56γ binding and the specificity of Apc1-loop^{500} to B subunits.

A Schematic diagrams of Apc1-loop^{500} constructs used (B). Same as Fig 1B, Apc1-loop^{500} (residues 515–584) was fused with maltose binding protein (MBP) at N-terminus and 6xHis at C-terminus. Conserved Cdk phosphorylation sites (SP/TP) are shown as S or T, respectively. The putative B56 binding region is shown in green. The alanine substitution mutations to three Cdk phosphorylation sites (3A) or to single point mutation S558A are shown.

B Binding assay using MBP-fused Apc1-loop^{500} fragments and B56γ using Apc1-loop^{500} WT or its derivatives (3A or S558A) in A. The bound proteins were analysed as in Fig 1C. The bar graph is quantification of bound B56γ. The intensities of MBP control were arbitrarily set to 1.0. Error bars, SEM from three independent experiments.

C Specific binding of Apc1-loop^{500} to B56γ. Apc1-loop^{500} WT or its derivatives (Δ11 or L557A/V560A) were incubated with the ^{35}S-labelled Flag-B56γ, Flag-B55δ or Flag-PR70 in anaphase extract supplemented with CycBΔ167 at 23°C for 1 h. The bound proteins were analysed as in Fig 1C. The bar graph is quantification of bound B subunits. The intensities of MBP control were arbitrarily set to 1.0. Error bars, SEM from three independent experiments.

Figure EV2. Mutant apo-APC/C complexes deficient in B56-APC/C interactions show less activity than WT APC/C.

A (left panel) The purified recombinant wild-type (WT) or B56 binding site mutant APC/C (1-Δ11) was incubated with APC/C-depleted (ΔAPC) interphase extract (Inter) or ΔAPC anaphase extract supplemented with CycBΔ167 (Ana) at 23°C for indicated times. The APC/C was recovered with Apc3 monoclonal antibody (AF3.1) beads, and the bound proteins were analysed by SDS–PAGE and immunoblotting with indicated antibodies. pApc1 (pS314/pS318) is a phospho-site-specific antibody. (right panel) Quantification of bound Cdc20. The intensities of WT control in interphase were arbitrarily set to 1.0. Error bars, SEM from three independent experiments.

B (left panel) Mutant apo-APC/C carrying mutations in Apc1 (1-L557A/V560A) binds Cdc20 at a lower level than WT APC/C. The recovered APC/C-Cdc20 complex used in Fig 2C was analysed by SDS–PAGE and immunoblotting with indicated antibodies. (right panel) Quantification of bound Cdc20. The intensities of WT APC/C control were arbitrarily set to 1.0. Error bars, SEM from three independent experiments.

C (left panel) B56 binding site mutant APC/C in Apc1-loop^{500} (1-Δ11) is less active in ubiquitylation assay than WT APC/C. The purified recombinant WT or 1-Δ11 APC/C was incubated with ΔAPC anaphase extract. The recovered APC/C-Cdc20 complex was subjected to ubiquitylation assay using ^{35}S-labelled cyclin B as a substrate. Samples were taken at indicated time points and analysed by SDS–PAGE and autoradiography. (right panel) Quantification of ubiquitylation assays. Error bars, SEM from three independent experiments.

D (left panel) The recovered APC/C-Cdc20 complex used in (C) was analysed by SDS–PAGE and immunoblotting with indicated antibodies. (right panel) Quantification of bound Cdc20. The intensities of WT APC/C control were arbitrarily set to 1.0. Error bars, SEM from three independent experiments.

 © 2019 The Authors

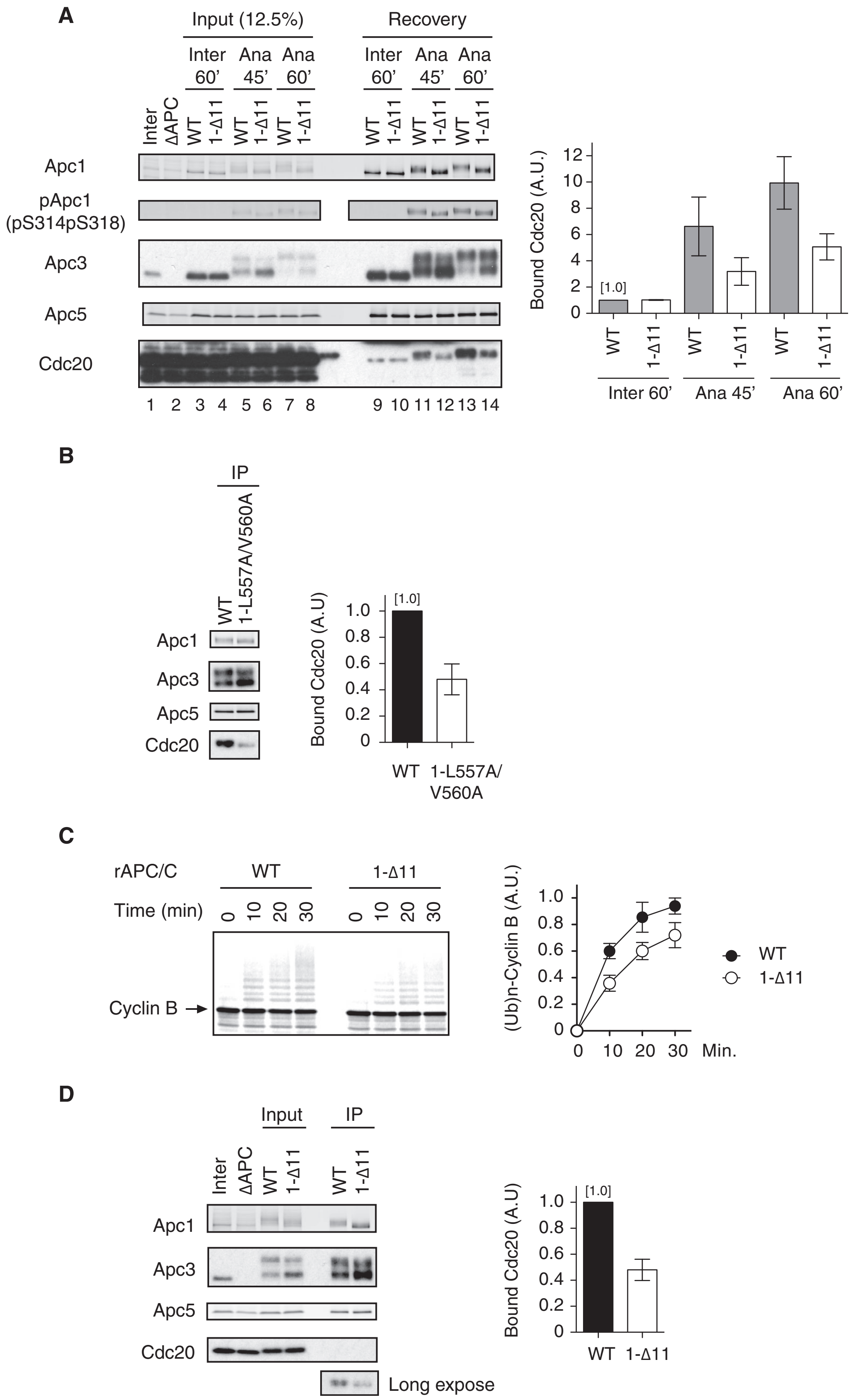
A
Input (12.5%)
Recovery
Inter 60'
Ana 45'
Ana 60'
Inter
ΔAPC
WT
1-Δ11
Apc1
pApc1 (pS314pS318)
Apc3
Apc5
Cdc20
1 2 3 4 5 6 7 8 9 10 11 12 13 14
Bound Cdc20 (A.U.)
[1.0]
B
IP
WT
1-L557A/V560A
Bound Cdc20 (A.U)
1-L557A/ V560A
C
rAPC/C
Time (min)
0 10 20 30
Cyclin B
(Ub)n-Cyclin B (A.U.)
Min.
D
Input
IP
Long expose

Figure EV2.

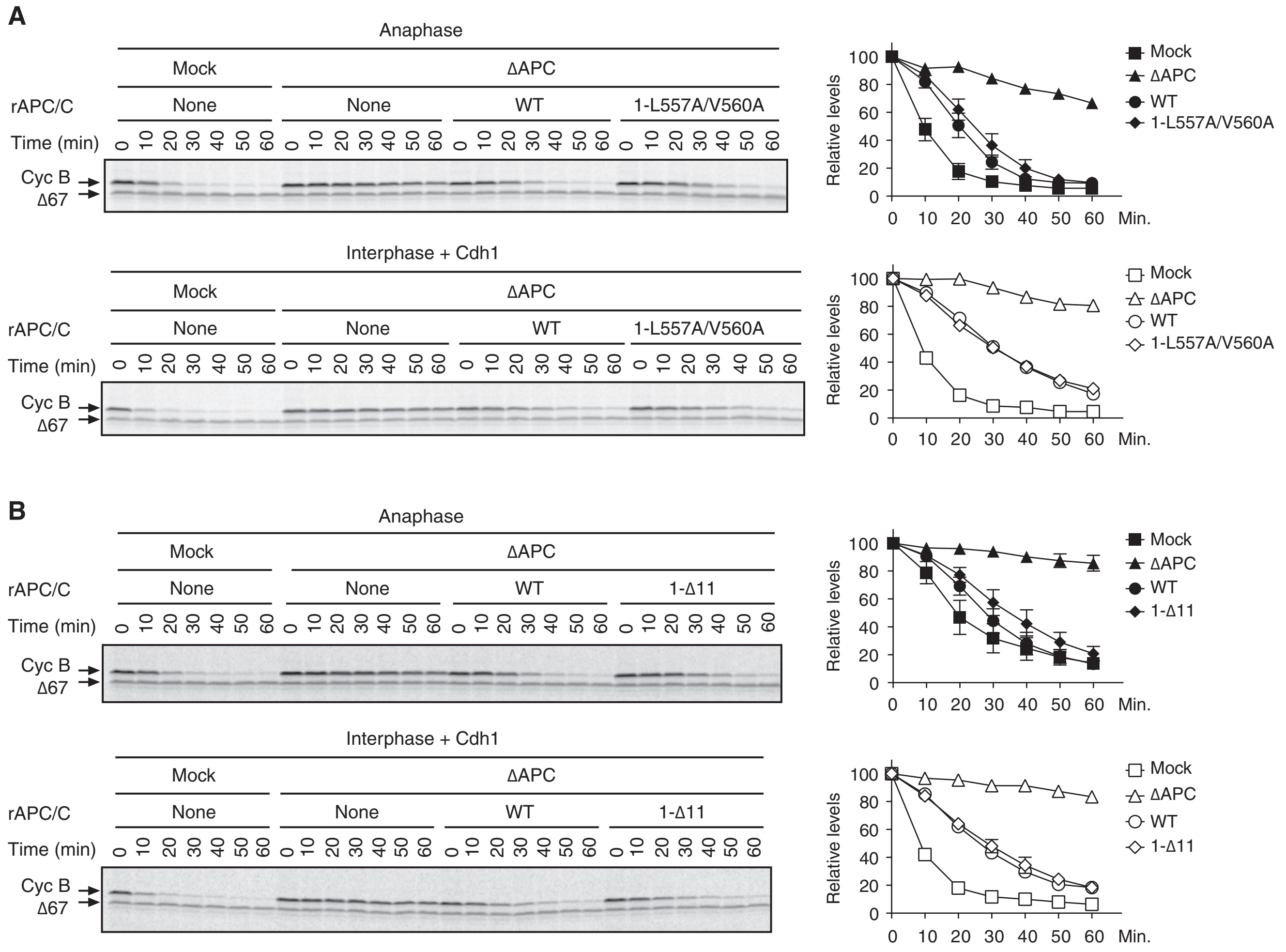

Figure EV3. Cyclin destruction assays using cell-free *Xenopus* egg extracts.

A (left panel) The B56 binding site mutant APC/C (1-L557A/V560A) is less active than WT APC/C in cyclin destruction assays in an anaphase-specific manner, whereas both activities are similar in interphase cyclin destruction assays. The purified recombinant WT APC/C or Apc1 mutant APC/C (1-L557A/V560A) was incubated with APC/C-depleted (ΔAPC) extract supplemented with CycBΔ167 (anaphase, upper panel) or with Cdh1 (interphase, lower panel) at 23°C. ^{35}S-labelled cyclin B and a version of cyclin B lacking the N-terminal 67 residues (Δ67, stable control) were used as substrates. Samples taken at indicated time points after addition of substrates were analysed by SDS–PAGE and autoradiography. (right panel) Quantification of cyclin destruction assays. Error bars, SEM from three independent experiments.

B (left panel) The activity of the B56 binding site mutant APC/C (1-Δ11) is less active than WT APC/C in anaphase cyclin destruction assay. The activity of recombinant APC/C (1-Δ11) was examined as in (A) (right panel) Quantification of cyclin destruction assays. Error bars, SEM from three independent experiments.

 © 2019 The Authors

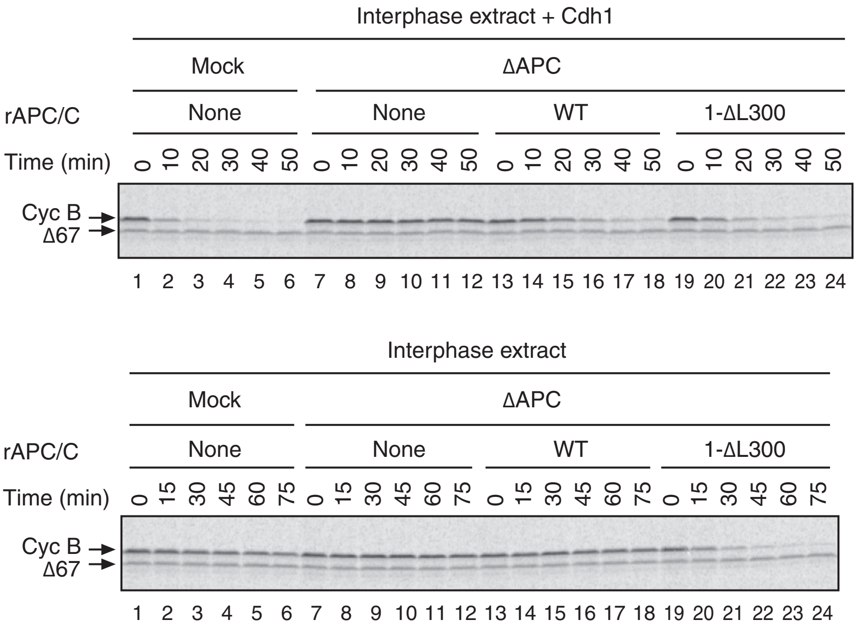

Figure EV4. Deletion of Apc1-loop^{300} activates the APC/C even in interphase extract.

In order to perform cyclin destruction assay, the purified recombinant WT APC/C or Apc1-loop^{300}-deleted APC/C (1-ΔL300) was incubated with ΔAPC interphase extract in the presence (upper panel) or absence (lower panel) of Cdh1 at 23°C. ^{35}S-labelled cyclin B and a version of cyclin B lacking the N-terminal 67 residues (Δ67, stable control) were used as substrates. Samples taken at indicated time points after addition of substrates were analysed by SDS–PAGE and autoradiography. 1-ΔL300 APC/C is activated by the endogenous Cdc20 and initiates cyclin destruction in the absence of Cdh1 in interphase (lanes 19–24, lower panel).

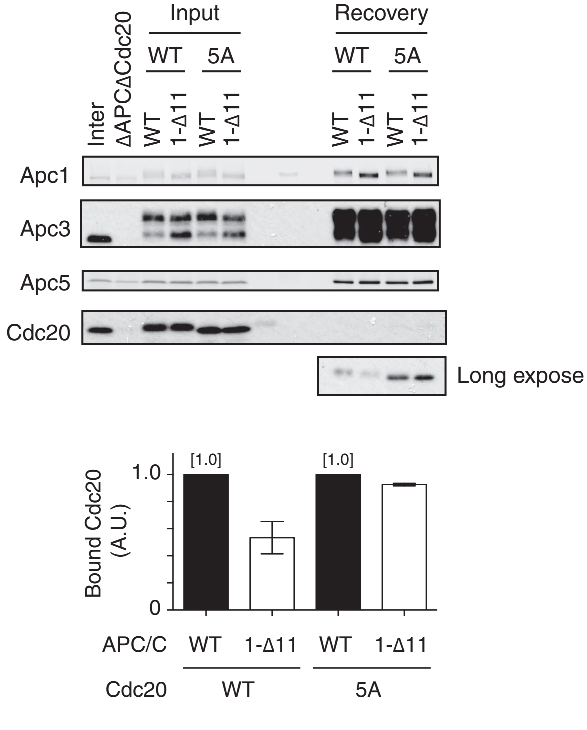

Figure EV5. Cdc20-5A efficiently binds to the B56 binding site mutant APC/C.

The purified recombinant WT APC/C or B56 binding site mutant APC/C (1-Δ11) was incubated with WT Cdc20 or non-phosphorylatable Cdc20 mutant (5A) in ΔAPCΔCdc20 anaphase extract at 23°C for 55 min. The APC/C was recovered with Apc3 monoclonal antibody (AF3.1) beads, and the bound proteins were analysed by SDS–PAGE and immunoblotting with indicated antibodies.

© 2019 The Authors
